# Statistical Rigor and the Perils of Chance

**DOI:** 10.1523/ENEURO.0030-16.2016

**Published:** 2016-07-14

**Authors:** Katherine S. Button

**Affiliations:** ^1^Department of Psychology, University of Bath, Bath BA2 7AY, United Kingdom

## Significance Statement

Concerns about the reliability and reproducibility of biomedical research have been voiced across several arenas. In this commentary, I discuss how a poor appreciation of the role of chance in statistical inference contributes to this problem. In particular, how poor scientific design, such as low statistical power, and questionable research practices, such as *post hoc* hypothesizing and undisclosed flexibility in analyses, yield a high proportion of false-positive results. I discuss how the current publication and funding system perpetuates this poor practice by rewarding positive, yet often unreliable, results over rigorous methods. I conclude by discussing how scientists can prevent being fooled by chance findings by adopting well established, but often ignored, methodological best-practice.

## 

There is increasing awareness of the problem of unreliable findings across biomedical sciences (Ioannidis, 2005). Many “landmark” findings could not be replicated (Scott et al., 2008; Begley and Ellis, 2012; Steward et al., 2012) and many promising preclinical findings have failed to translate into clinical application (Perel et al., 2007; Prinz et al., 2011), leading many to question whether science is broken (Economist,2013). Central to this problem is a poor appreciation of the role of chance in the scientific process. As neuroscience has developed over the past 50 years, many of the large, easily observable effects have been found, and the field is likely pursuing smaller and more subtle effects. The corresponding growth in computational capabilities (Moore, 1998) means that researchers can run numerous tests on a single dataset in a matter of minutes. The human brain processes randomness poorly, and the huge potential for undisclosed analytical flexibility in modern data-management packages leaves researchers increasingly vulnerable to being fooled by chance.

## The role of chance in statistical inference

Researchers cannot measure an entire population of interest, so they take samples and use statistical inference to determine the probability that the results they observe represent some underlying biological truth. Samples vary in how closely they represent the true population, and this variation is inversely related to sample size. The probability of drawing correct inferences depends on the size of the sample, the size of the effect under investigation, the significance threshold for claiming an effect (alpha, typically 5%), and the statistical power of the test (1− beta). These four parameters are mathematically coupled so each can be calculated from the remaining three; a mathematical principle which proves useful in studying various forms of bias in a given literature (Button et al., 2013).

In terms of a single statistical test, there are two main ways scientists can be fooled by chance. They can commit a type I error and falsely reject the null hypothesis when it is in fact true (ie, a false-positive decision), or they can commit a type II error by failing to reject the null hypothesis when it is in fact false (ie, a false-negative decision). In a third way, they can overestimate/underestimate the magnitude of a genuine effect.

Statistical power determines the probability of correctly rejecting the null hypothesis. Thus, power is related to the rate of true-positives and inversely to the rate of false-negatives. The lower the statistical power, the lower the chances of detecting genuine effects. The significance or alpha criterion, typically 5%, sets the probabilistic threshold for rejecting the null hypothesis, and determines the probability of committing a type I error and making a false-positive decision.

A common misconception is that that the risk of making a false-positive decision is solely determined by the alpha criterion, and that the only risk associated with insufficient power is missing genuine effects. However, if the pre-study odds of a hypothesis being true (the ratio *R* of “true effects” over “null effects” in the scientific field) is taken into account, then statistical power is also related to the probability of a positive result being true-positive, this is known as the positive predictive value of the test (PPV). The PPV can be calculated for given values of statistical power (1 – β), pre-study odds (*R*), and type I error rate (α), using the formula PPV = ([1 – β] × R) / ([1− β] × R + α). The formula shows that, for studies with a given pre-study odds *R*, and a given type I error (for example, the traditional *p* = 0.05 threshold), the lower the power, the lower the PPV (Button et al., 2013). Confirmatory or replication studies testing pre-specified hypotheses have higher pre-study odds as the weight of previous evidence or theory is behind them. The pre-study odds are lower for exploratory studies that make no prior predictions, leaving the findings more open to chance. Combining low statistical power with low pre-study odds has dire consequences for PPV. Suppose we are working in a highly exploratory field where in 90% of cases the null hypothesis is true. If we conducted 1000 studies with alpha set at 5%, 45 (ie, 5% of the 900 studies where the null hypothesis is true) would be expected to yield false-positive results. If average power were 80%, 80 studies would be expected to yield true-positive results (ie, 80% of 100 genuine associations), meaning the probability that any single positive result was true is 64% (PPV = 0.64). However, if the average power were only 20% then this probability would drop to 31% (PPV = 0.31), as the proportion of true-positive findings would drop from 80 to 20, whereas the number of expected false-positives (ie, 45) would stay the same (Sterne and Davey Smith, 2001).

Even if the researcher is lucky enough to make the correct inference, they may still be fooled by sampling variation, and underestimate/overestimate the size of the true effect (or even in some cases find a significant effect in the opposite direction; Gelman and Carlin, 2014). These errors of magnitude are more likely in smaller studies where the results are more variable. As small studies often have insufficient power to detect the genuine effect size, only those small studies, which yield results that by chance grossly overestimate the true effect size, will reach statistical significance. This is often referred to as the winner’s curse, as the researchers are winners to have found a positive (and thus potentially more publishable) result. However, they are cursed as their result is a grossly inflated estimate (Button et al., 2013)

Designing studies with sufficient statistical power (typically considered 80% or more) is therefore crucial to reduce the chances of making false inferences. However, there is a preponderance of small underpowered studies in many research fields. The median statistical power in the neurosciences is estimated at close to 20% (Button et al., 2013). This has important consequences for the veracity of research findings. Studies with power this low will on average miss 80% of genuine effects, whereas the probability of a positive result being true (PPV) is only 31% for exploratory research (assuming pre-study odds = 0.11) rising to 80% for confirmatory studies (pre-study odds = 1). Furthermore, effect estimates for positive results would be expected to be inflated by ∼50% (Button et al., 2013).

## Fooled by randomness and a talent for self-deception

The human brain is particularly poor at understanding the play of chance in everyday events. Random events which fit with current goals or beliefs are often interpreted as important or causal (eg, a profit trading stocks and shares is due to talent), whereas events that contradict are quickly dismissed as being irrelevant or due to chance (a trading loss is due to bad luck; Taleb, 2007). Far from the objective ideal, scientists are invested in the outcome of their experiments, hoping to find support for theories both for the simple pleasure of having one’s expectations confirmed, and for the positive results that lead to publications and career progression. Despite our best efforts, the brain automatically favors processing information in accordance with our own goals and desires, leaving us poorly positioned to draw accurate inferences based on probability. Put simply, statistical inference is simply not intuitive.

To compounds matters further, insufficient or inadequate statistical training means that many neuroscientists, including senior investigators, may lack basic statistical literacy. This lack of statistical savvy with the speed and power of modern computation leaves researchers more vulnerable than ever to fooling themselves (Nuzzo, 2015). Researchers can easily explore multiple analytical pathways, such as removing an outlier, transforming a variable, collecting more data, switching outcome variables, adding or removing covariates, until they happen upon a significant result. Such flexibility in analysis is perfectly acceptable as long as it is transparently reported so it can be appropriately accounted for when drawing inferences. However, whether deliberately, due to unconscious bias, or due to statistical illiteracy, researchers often forget about the unsuccessful paths reporting only those leading to statistically significant results (Simmons et al., 2011). There is good evidence that such undisclosed flexibility in analysis is commonplace, both from surveys of research practice (John et al., 2012), and by the incredible 85–90% of neuroscience/psychology/psychiatry papers claiming evidence for an a priori hypothesis (Fanelli, 2010b). Either a high proportion of researchers are researching redundant questions, where the answer is already known, or they are exploring their data to find a significant result and then hypothesizing afterward (Simmons et al., 2011).

## Current incentive structures perpetuate poor practice

Scientific practices that fail to account for chance findings yield unreliable results, yet they persist for a variety of reasons. Scientists are under increasing career competition. Over the past 30 years, the number of faculty positions in the US has remained relatively constant, but the number of PhDs awarded has increased dramatically (Schillebeeckx et al., 2013). The biggest predictor of academic success is the number of first author publications, followed by the impact factors of the corresponding journals (van Dijk et al., 2014). Unfortunately, this “publish or perish” culture, in the presence of the long-standing publication bias for novelty and positive results, may incentivize running multiple small studies measuring multiple outcomes. As described above, such practice combined with flexible analytical procedures (Simmons et al., 2011), can generate a large number of positive results, although most will either be false-positive or inflated (Button et al., 2013). These positive results are often incorrectly reported as confirmatory (John et al., 2012), are disproportionately rewarded with publication (Rosenthal, 1979), potentially leading to grant funding and career advancement (van Dijk et al., 2014). Indeed, the degree of bias or inflation in reported effects correlates (albeit weakly) to the impact factor of the publishing journal, with highly inflated results from small studies being rewarded with publication in some of the highest impact journals (Munafò et al., 2009). Furthermore, competitive research environments increase the proportion of studies reporting positive results (Fanelli, 2010a), providing evidence that current incentive structures perpetuate poor practices.

## Solutions

Solving these issues requires a systemic shift in both thinking and practice. Solutions include preregistration of study protocols (Dickersin and Rennie, 2012), transparent reporting of methods and results (Rennie, 2001; Simera et al., 2010), and designing studies with sufficient statistical power (Button et al., 2013). Better education and training in research methods and statistics are vital to equip neuroscientists with the skills required to deliver rigorous research, and to better peer-review the work of their colleagues. However, with the complexity of data in some fields of neuroscience and the advancement of modern statistical techniques, it may be time for a move toward working in multidisciplinary teams which include a statistician.

Perhaps the most powerful way of preventing scientists from fooling themselves or their colleagues into false interpretations of chance findings is transparent reporting. Transparency can be facilitated by public registration of study protocols and analysis plans before data is collected. This creates an audit trail, and the clear differentiation between confirmatory tests of a priori hypotheses, and *post hoc* explorations of data. Statistics should also be reported transparently so that others can use the data for power calculations or meta-analysis. Means and standard deviations, as well as effect sizes and confidence intervals should be routinely reported in addition to test statistics and *p* values. Reporting actual *p* values rather than *p* </> 0.05 protects against the temptation for rounding errors (John et al., 2012). Where ethics and participant consent permits, data should be made open-access.

Blinding study personnel to experimental conditions wherever possible is also essential for reducing the impact of unconscious bias, particularly during data collection (Macleod et al., 2008). Blinded data analysis can protect against asymmetrical data-checking (where researchers check unexpected or null findings more thoroughly for errors than findings that fit with their expectations), *p*-hacking (exploring data until a significant result is found), and other biased decisions about data-cleaning (Nuzzo, 2015).

## Aligning career incentives with robust science

Conducting more rigorous research has implications; better powered studies require more resources, take longer to run, and often yield more conservative results. However, fewer, more conservative papers could leave a scientist at a career disadvantage in the current system. To prevent this we need systemic change, realigning the incentive structures for career advancement with rigorous methods. Fields, such as clinical trials and human genome epidemiology, have arguably led the way in terms of trial registration and transparent reporting (Rennie, 2001; Simera et al., 2010; Dickersin and Rennie, 2012), and large-scale collaborative consortia with extensive replication (Munafò and Flint, 2014), respectively.

However, change is happening in neuroscience. Funders and publishers are implementing new funding and publishing requirements and initiatives (Landis et al., 2012; Chambers, 2013; Nature, 2013; Munafò et al., 2014). These include checklists for minimum standards of reporting to improve transparency (eg, https://bmcneurosci.biomedcentral.com/submission-guidelines). Furthermore, based on the Organization for Economic Co-operation and Development (OECD) assertion that publicly-funded research data are a public good, produced in the public interest, and thus should be openly available as far as possible, many funders and publishers now require data and research resources to be made publically available (eg, http://www.mrc.ac.uk/research/research-policy-ethics/data-sharing/policy/).

Funders can support high-quality research, by funding larger studies, which may involve collaboration across multiple research groups. However, even in the absence of substantial grant funding, researchers can find innovative ways to maximize research resources and boost power through collaboration (Button et al., 2016; Schweinsberg et al., 2016). There are also numerous researcher-led initiatives for improving transparency and replication (Kilkenny et al., 2010; Open Science Collaboration, 2015), including open source science initiatives to share knowledge, resources, and even crowd-source research projects (eg, http://www.theopensourcescienceproject.com). The benefits for collaborative studies are far reaching. Results obtained from multiple laboratories are often more generalizable, and the need to share data and harmonize methods necessitates transparency in reporting, whilst expediting the development of optimal research procedures. Successfully adopting robust methods will inevitably change the nature of the evidence base, and we should be prepared for this. In the clinical trials literature protocol preregistration and specifying primary outcomes is often mandatory, and the number of trials finding in favor of a new drug is around 50–60%, close to the point expected by clinical equipoise (Djulbegovic et al., 2013). Clinical trials are arguably the most confirmatory type of research, resulting from years of preclinical findings and early-phase trials. By comparison, the majority of neuroscience research will be much more exploratory. The current rate of 85–90% of neuroscience papers confirming a priori hypothesis (Fanelli, 2010a) is unsustainable. Successful implementation of rigorous methods would be expected to more than half of this rate. We should also expect unintended consequences; for example, too great a swing toward confirmatory research might stifle innovation and hypothesis generation. However, the growth in meta-research (that is, science on science) provides a powerful means of measuring these changes, allowing us to monitor our progress toward a more reliable evidence base.

## Synthesis

The decision was a result of the Reviewing Editor Margaret McCarthy and the peer reviewers coming together and discussing their recommendations until a consensus was reached. A fact-based synthesis statement explaining their decision and outlining what is needed to prepare a revision is listed below. The following reviewers agreed to reveal their identity: Suresh JESUTHASAN, Evelyn Schlenker

this is an excellently written and timely review on the importance of chance and its interpretation in statistical analyses. Two expert reviewers are enthusiastic about the significance of the review and the many important ideas presented. They offer the following items as topics that were either missed or not as well represented as they could be:

1) In considering causes, the author may wish to also consider the possibility that scientists can fall victim to wanting to have their expectations met: a major reason for stress is the mismatch between expectation and reality, and a great source of pleasure is the converse. This is separate from external pressures to perform (line 91).

2) In Page 7, line 164, it would be informative to elaborate what the unintended consequences are (eg, are larger studies aiming towards higher statistical power always going to be beneficial or is there a diminishing return?). In the same line, the benefits/explanation of meta-research is unclear.

3) The benefits for collaborative and open source studies could be emphasised. At present, these are seen only in the framework of increasing statistical power.

4) While self-deception and pressure to publish positive findings has led to false positives being reported, a lack of knowledge of appropriate statistical analysis may also be a cause. The drive to use sufficiently powered studies is relatively new in experimental neuroscience, and may not have penetrated into neuroscience teaching at the undergraduate and graduate levels. In thinking about solutions, one could include steps to remedy this.

5) Neuroscience is still largely biased toward finding whether p is less than 0.05. Researchers rarely report effect sizes and confidence intervals, but show mean and standard deviations that do not reflect the variations in the underlying data accurately and make inferences and replications difficult. This could be elaborated on Page 6, line 128, transparent reporting of methods and results).

6) Two items that are not addressed are the lack of investigator statistical literacy and the fact that many journals have more vigorous requirements (checklists) to report experimental designs and statistical analysis.

7) nothing is said about the duty of journal reviewers (sometimes statistical specialists) in determining the rigor of studies.

The reviewers also had some specific editorial suggestions and comments:

1)Page 6, line 127, "The" should be removed in the beginning of the sentence

2) Page 5, line 99, Add "to" after "due".

3) The paper by Vesterinen et al., 2011 (lines 26-27) does not really provide evidence for the statement "Despite likely decreasing effect sizes, average statistical power of studies has remained relatively constant." It provides a great deal of other important points.

4) Discussion that begins on line 43: "In terms of the results of statistical test outcomes there are three main ways scientists can be fooled by chance." Why not start with a Type 1 error, then type 2 error and then errors due to estimating the magnitude of the effect. ? this is an editorial suggestion and your choice as author to determine

5) Lines 54 and on: A definition of PPV and how it related to power and alpha would be useful here. The Button 2013b article does an excellent job defining this factor. If this paper is read by experts in statistics, they may be familiar with this concept, others may not be.

6) Line 93: Defining "undisclosed flexibility in analysis" and giving an example would be instructive.
